# INCREASED SUPPORT FOR OLDER VETERANS AND THEIR CAREGIVERS USING TELEHEALTH SERVICES

**DOI:** 10.1093/geroni/igad104.2311

**Published:** 2023-12-21

**Authors:** Bertha Flores, Lyda Arevalo-Flechas, Rozmin Jiwani, Enelda Romo, Gabriela Zaragoza, Becky Powers, Sandra Sanchez-Reilly, Sara Espinoza

**Affiliations:** University of Texas HSC at San Antonio, San Antonio, Texas, United States; South Texas Veterans Health Care System, San Antonio, Texas, United States; UT Health San Antonio, San Antonio, Texas, United States; South Texas Veterans Health Care System, San Antonio, Texas, United States; South Texas Veterans Health Care System, San Antonio, Texas, United States; South Texas Veterans Health Care System, San Antonio, Texas, United States; South Texas Veterans Health Care System, San Antonio, Texas, United States; South Texas Veterans Health Care System, San Antonio, Texas, United States

## Abstract

Five million Veterans live in rural areas in the U.S., and about half of those live in the South. Rural Veterans are more likely to be older and have a disability compared with non-rural Veterans. In Texas, Veterans comprise seven percent of the adult population with a median age of 50 years, of those 17% live in rural areas where access to care is a concern for many Veterans and their families. Technology-based services could help close this gap. The GRECC Connect program is one way to reach rural Veterans and their caregivers. GRECC Connect is a multi-disciplinary virtual geriatrics program that provides support for providers to help manage older, rural Veterans through telemedicine. Patient Tele- consultations follow the Age-Friendly Geriatrics 5 M’s framework (Medications, Mentation, Mobility, Multi-complexity, and what Matters most). As a demonstration project at the San Antonio GRECC, we partnered with the Geriatric Extended Care Services (GEC), and the Dementia Caregiver Program (DCP) to educate and support caregivers. We also partnered with the education department to conduct clinic outreach. In Fiscal year 2022, we served 72 Veterans, 66 of those had a caregiver, and the majority (51/66, 77%) were enrolled in the DCP. In Q4 (17/17, 100%) of caregivers were enrolled in the DCP. In addition, Veterans were referred to 248 additional services including palliative care and saved 5,934 miles. The GRECC Connect program decreases barriers experienced by older rural Veterans including transportation, assistance to navigate VA system and access to specialty geriatric and palliative care.

